# The anatomy theatre and the slaughterhouse: emotion, vivisection, and the disciplines of medicine in the experimental practices of Charles Bell

**DOI:** 10.1017/mdh.2026.10058

**Published:** 2026-07

**Authors:** James Bradley

**Affiliations:** School of Historical and Philosophical Studies, University of Melbourne, Australia

**Keywords:** anatomy, physiology, vivisection, nervous system, emotions, Charles Bell

## Abstract

This article examines Charles Bell’s experimental practices by drawing historiographical attention away from the priority disputes over the spinal nerve functions for which he was most famous. I argue that Bell’s primary research interest was the expression of emotions. To this end, he developed a programme of vivisection that explored the underlying mechanisms of emotion. However, this also resulted in a profound contradiction between his experimental practices and his worldview – conducting painful experiments on beloved animals despite moral revulsion towards animal experimentation. This opens up three interconnected areas. Firstly, it allows an exploration of disciplinary identity in medicine, particularly the way that disciplines demanded specific practices and behaviours. Secondly, vivisection more generally required methods and ethics that opposed the growing anti-cruelty voice. Here, a combination of animal choice and the importation of techniques from the slaughterhouses was critical. Thirdly, vivisectors navigated a complex emotional landscape between their professional obligations and broader cultural sensibilities. These three areas are linked together using Boddice’s concept of moral economies, the affective frameworks that structured feelings. Particularly important were the sentimental and Romantic economies, both of which impacted Bell and his research. At the same time, Bell always struggled to reconcile the tensions between his disciplinary identity and his sentimental and Romantic beliefs, ultimately leading him to abandon experimentation after his assistant John Shaw’s death. I conclude by identifying the guarantees provided by character for licensing ostensibly cruel behaviours, thus allowing for the maintenance of probity within competing moral economies.

## Introduction

There has been much sound and fury over whether Charles Bell discovered the motor and sensory roots of the spinal nerves or, indeed, the related but lesser-ranked discovery of the functions of the trigeminal and facial nerves.^1^ So much, in fact, that some of the more interesting things about Bell and his experimental practices have been missed. For example, the motor and sensory nervous functions were, at best, contingent on his investigations. Rather, he was concerned with the dynamic interplay of the nerves and emotional expression, speculating the existence of a discrete respiratory nervous system that connected the heart and lungs to the face, allowing the development of feelings into emotions and emotions into expression. This discovery was eclipsed by his lengthy and largely successful campaign to claim discoveries he had not made. But because historians have focused on the priority disputes, with the assumption that the separate functions were Bell’s *telos*, they have not only missed the true cultural import of his original system (a foundation of the modern psychology of emotions), but also the extent to which his culture and worldview determined his experimental practices.^2^

Bell’s love of animals provides the starting point. As many commentators have noted, and as Bell’s letters confirm, he had a moral loathing of vivisection. His voluble utterances against the ‘cruelty and indifference’ of the French experimental physiologists were frequent and forthright,^3^ and yet descriptions of Bell’s experiments, particularly those by John Shaw (his brother-in-law and principal assistant), reveal their wide-ranging use, often performed on species for which he had inordinate affection.^4^ Bell, for example, loved donkeys, drawing them with evident delight, including a sketch made the day before his death.^5^ He tenderly referred to them in the Scots dialect as ‘cuddies’, yet they were crucial experimental subjects.^6^ He also had deep attachments to companion animals, including his two Skye terriers, Striach and Feoch. But live dogs likewise found their way into Bell’s anatomy theatre at the Great Windmill Street school, where many of his experiments were conducted.^7^ Why did Bell vivisect when he repeatedly insisted that anatomy alone was fit for the purpose of discovery? Equally, why did he repeatedly vivisect animals for which he had affection when he held significant moral qualms about the ethics of this practice?

Prising apart the contradiction between Bell’s experimental practices and his ethical worldview creates a space for exploring three overlapping historiographical areas. Firstly, the functions of disciplinarity in medicine and how it shaped practice. Here, I will provide nuance to the standard portrayal of Bell as an anatomist whose experiments were nothing more than an afterthought. Guerrini, for example, suggests that he criticised François Magendie, his main competitor for priority, on methodological grounds (i.e. anatomy over physiology) rather than ethics.^8^ This was true – but only up to a point. Bell was remarkably consistent about anatomy’s precedence over physiology, arguing thus in 1819 *before* his discoveries were announced and similarly in 1834, many years *after.* ^9^ But – and this is missed by Guerrini – it was exactly because he *was* first and foremost an anatomist that he had a disciplinary commitment to vivisection, irrespective of his profound moral qualms. Indeed, he was possibly the last great representative of Andrew Cunningham’s ‘old anatomy’, a discipline that demanded experimentation as its final step.^10^

Secondly, Bell provides a case study for understanding the broader practice and culture of vivisection. For while he despised the emerging discipline of experimental physiology, he benefitted from several innovations that were fundamental to the new discipline as practised in Britain.^11^ A.P. Wilson Philip, in particular, promoted techniques that aimed to make vivisection more humane and therefore immune to the fury of anti-vivisectionists. These had been developed in a dialogue between the slaughterhouse and the anatomy theatre. Bell adopted these techniques whenever vivisection would lead to the animal’s death. However, his most commonly reported experiments were those upon the facial nerves, conducted without recourse to pain mitigation. While these were less invasive and rarely fatal, they were performed on agonised and fully conscious subjects whose survival was crucial because the experiments were designed to observe the effects of nerve division on the expression of emotion.

Thirdly, describing the experimental milieu brings us into the affective universe of vivisectors, particularly the emotional tensions between disciplinary commitments and the wider culture beyond the anatomy theatre. Here I will use Bell’s experimental practices as a micro-history to explore concepts drawn from the history of emotions, particularly the social and cultural frameworks that shape the expression of emotion. Attention will be paid to Rob Boddice’s model, which builds upon the work of William Reddy.^12^ Both attempt to steer a course between the Scylla of cultural constructionism (emotions are specific, even unique, to particular cultures) and the Charybdis of biological essentialism (emotions are universally experienced by all members of *Homo sapiens* irrespective of time or place). Reddy presented a ‘biocultural’ *via media* governed by ‘emotives’ that translate raw feelings into emotions through language, allowing the subject to express their sentiments in culturally appropriate ways. ‘Emotives’ are grounded in specific ‘emotional regimes’ that laid down the possibilities and boundaries of emotional expression.^13^ While Boddice was happy to accept the biocultural thrust of Reddy’s theory, including the function of emotives, he proposed that the emotional regime concept is too blunt, arguing instead for moral economies. Unlike Reddy’s regimes or Barbara Rosenwein’s ‘emotional communities’, moral economies work alongside, against, or within each other (like nested Russian dolls). Experimental physiologists, for example, might be part of a larger sympathetic moral economy, sharing with their opponents a belief in the importance of compassion, but their disciplinary moral economy, nested within the larger economy, implied that their laboratory-based cruelty was a more robust form of compassion than that of their anti-vivisection critics. Boddice’s model effectively explains what Paul White has called ‘the divided self’ of vivisectors (animal lovers who destroyed what they loved), but also points to the possibility that the emotional negotiation between the two conflicting economies might be partially or wholly unsuccessful.^14^

This article, therefore, argues that vivisection not only operated within specific moral economies that were often linked to disciplinary identity, but also that the practice conflicted with the sentimental economies of wider culture. This demanded navigation on the part of all vivisectors and, if possible, reconciliation between the conflicting demands of these economies. In Wilson Philip’s case, it drove him to employ slaughterhouse techniques and embed them within something approaching an ethical code, while for Bell it would have a significant impact upon his experiments and their findings.

## ‘Old anatomy’ versus new physiology in the practice of vivisection

Using Amariah Brigham’s contemporary list (1840), Edwin Clarke and L.S. Jacyna explored the methods used by nineteenth-century medical scientists to uncover the hidden workings of the nervous system: gross anatomy (human); experiment (vivisection); comparative anatomy (exploring the structural analogies and homologies between different species, including humans); developmental anatomy (especially embryology); anatomical-pathology (clinical observation followed by post-mortem); and finally, microscopy.^15^ Although some of these were uncontroversial, both gross anatomy and vivisection, two of Bell’s four methods, caused considerable unease. Much has been written about anatomy’s collision with wider cultural beliefs about the body and soul.^16^ Less has been said about vivisection, at least with regard to the first half of the nineteenth century; but what has been said depicts a controversial practice raising popular anxieties at a time when cruelty towards animals was being challenged by reformers inside and outside of medicine.^17^

The concerns about vivisection expressed by anti-cruelty campaigners led to strategies designed to mitigate the suffering caused by vivisection, albeit by causing death. As we will see, the physician and physiologist A.P. Wilson Philip was especially attuned to long-standing concerns with vivisection, while actively promoting solutions.^18^ The focus of this section, however, is on the relationship between experimentation and the disciplines of medicine as far as it shaped Bell’s experiments. Cunningham’s work is particularly useful for mapping out the connections between vivisection and disciplinarity in medicine. He rebuts the received historiographic view that to conduct vivisection was to be a physiologist. This, he argues, is an anachronism: the collapse of vivisection into physiology is an artefact of experimental physiologists’ desire to bolster their emergent and, as yet, unstable identity. But, for most of the long eighteenth century, vivisection was integral to anatomy, not physiology. Eighteenth-century physiology was a rational pursuit, more akin to scholasticism than experimentalism. The ‘old physiology’, as Cunningham branded it, focused on ‘why’ questions – explaining the body’s functions by imposing the physical or chemical theories of the day upon the ‘what’ and ‘where’ questions that had been answered by anatomy. Anatomists answered the ‘what’ and ‘where’ questions using anatomical dissection to deduce function, followed by vivisection for testing those deductions.^19^

During the post-revolutionary period in France, experimental physiology overturned the old speculative and physician-based physiology, making vivisection the primary method for functional discovery.^20^ By systematically interrogating live animal bodies, it usurped anatomy’s position as arbiter of structure and function. It incrementally explored the body through the oft-repeated and always localised obliteration of individual parts until the specific function under exploration had been isolated and identified. According to Cunningham, Bell’s competitor Magendie was the father to the new discipline, although Lesch suggests that its birth was more complicated, giving a critical role to the emergence of the anatomical-pathological method as an immediate precursor.^21^

In the British context, historiography has emphasised the rarity of vivisection, while also arguing that experimental physiology barely took hold before the 1870s – a portrait that remains largely unquestioned and perhaps overly influenced by the Royal Commission on the Practice of Subjecting Live Animals to Experiments for Scientific Purposes (1875) and its aftermath.^22^ Indeed, beyond Cunningham, the knotty relationship between vivisection and the disciplines of medicine is largely unexplored, while the early history of British experimental physiology is often reduced to the lonely and heroic figure of Marshall Hall. Thus, most historians have struggled to identify the disciplinary commitments of practitioners of vivisection and the relationship it had to ‘old anatomy’ and ‘old physiology’. Geison, for example, includes Bell in a short list of English-based, Scottish-educated physiologists illustrating the influence of Scottish medical education on British physiology; the underlying assumption being that to practise vivisection was to be a physiologist.^23^ As I will show, while both Bell and the new experimental physiologists used vivisection, there was a gulf between the underlying logic of their experiments and the actual experimental practices themselves. I will now illustrate this point by comparing Bell with Wilson Philip.

Wilson Philip had been an experimentalist from his earliest days as a physician at Worcester. He had published in areas typical of a late Georgian physician, including an analysis of Malvern’s mineral waters,^24^ but had also shown an interest in experimentalism when studying the nature of fevers and their treatments.^25^ Later, he would investigate the relationship between the nervous system and digestion.^26^ His most significant work was on the connection between the nerves and the heart. Commencing his experiments on this subject around the same time Bell was investigating the connections between the brain and nervous system, which would result in his privately printed pamphlet *Idea of a New Anatomy of the Brain* (1811),^27^ Wilson Philip would eventually read three papers at the Royal Society. Later, these were combined with a translation of the National Institute of France’s report on Julian Legallois’s experiments into *An Experimental Inquiry into the Laws of Vital Function.* ^28^

The *Experimental Inquiry*, running to three editions, was the epitome of early nineteenth-century English experimental physiology. The agenda for Wilson Philip’s experiments was set by the longstanding question of whether the heart’s action was a function of properties inherent in its tissues or whether it received its power from another source (for example, the nerves or vascular system). Albrecht von Haller had argued a combination of the two: upon removal from the body, it remained active for a short period, its excitability the result of its natural irritability combined with the vital powers of the vascular system. Following a lengthy series of experiments, Legallois claimed to have overturned this view, concluding ‘that by the destruction of the whole or cervical part of the spinal marrow, the action of the heart is immediately so debilitated, that it is no longer capable of supporting the circulation’.^29^

Notwithstanding the National Institute’s imprimatur, Wilson Philip remained sceptical of Legallois’s findings. Initial experiments had revealed several theoretical holes, so he designed a new and rigorous set of experiments to test his point:

A rabbit was deprived of sensation and voluntary power by a stroke on the occiput. When the rabbit is killed in this way, the respiration immediately ceases; but the action of the heart and the circulation continue, and may be supported for a considerable length of time by artificial respiration.^30^

The rabbit was then disassembled: ‘the spinal marrow was laid bare from the occiput to the beginning of the dorsal vertebrae’, but the heart still beat strong, and continued to do so as long as artificial respiration was kept up. Next, the brain was removed, ‘so that no part of the nervous system remained above the dorsal vertebrae’. The heart continued beating as long as respiration occurred. But if respiration ceased, ‘the ventricles ceased to beat about half an hour after the removal of the brain’. Even then, renewed respiration might revive the heart’s activity.^31^

Wilson Philip’s experiments left a trail of dead rabbits and frogs in their wake (he estimated that 130 rabbits had been destroyed, although there was no tally of frog deaths).^32^ All the experiments involved snipping, cutting, the partial or wholesale destruction of brains and nerves, and the addition of poisonous substances here and there. The experiments became more complicated as each of Legallois’s conclusions was tested and then modified or repudiated. And so, the experiments went remorselessly on, with ever more complicated ways of inducing paralysis and death, while ponderously procuring knowledge. At times, the description of experiments was interrupted by lengthy discussions of the findings of other experimentalists, including those by William Clift,^33^ John Hunter’s erstwhile assistant and conservator of the Royal College of Surgeons, and the occasional but important modification of Legallois’s experimental techniques.^34^

If Legallois was a ‘new physiologist’, so too Wilson Philip. He was part of a group who may or may not have identified as experimental physiologists but definitely adopted many of its practices and much of its logic. A list of English-based practitioners would include Marshall Hall as its prime representative, but also James Blundell, Samuel Broughton, James Hope, Herbert Mayo (at least until the mid-1820s), Charles Hastings, C.J.B. Williams, and Wilson Philip himself. The circle expands with the inclusion of sympathisers and supporters, individuals like William Sharpey, who taught the new science but did not practise it, or others like Benjamin Brodie, who had engaged in experimentation but had largely abandoned it in favour of teaching and service to societies and colleges.

As with French experimental physiology, the vivisections performed by Wilson Philip and other early British experimentalists aimed to describe the dynamic connections within the living body by the selective destruction of its parts. In this way, function could be isolated – observing, for example, the effects of cutting a single nerve or ablating a portion of the brain. There was a specific logic at play. The experimental physiologist started with a question, often originating in recently conducted research, that could only be answered by a minute analysis of function. As a practice, therefore, it was the embodiment of the ‘analysis:synthesis’ style of reasoning identified by James Elwick: the analytical breaking down of the body into its smallest constituent parts followed by synthesis, the development of a theory that demonstrated the co-functioning of those parts.^35^

Elwick identified Bell as a practitioner of ‘analysis:synthesis’ and, therefore, brought him into the orbit of experimentalists like Wilson Philip.^36^ However, while on the surface his techniques appeared similar to Wilson Philip’s, the underlying logic was profoundly different. For example, Wilson Philip was in dialogue with the French experimental school. The questions he asked directly derived from Legallois’s findings, and the methods he used to answer them were identical to those of the French physiologist.^37^ Bell, however, claimed that he was driven by deductions from his anatomical research, occasionally buttressed by pathological analogy. Time and again he reiterated this point, distancing himself from the new physiology while rejecting the entire body of knowledge produced by the French school.^38^ However, if we strip away the rhetoric and reconstruct Bell’s research programme, it becomes clear that it was driven by a set of *a priori* judgements, which shaped the conduct of the experiments themselves.

Nowhere are these issues better seen than in Bell and Shaw’s attempts to discredit the work of Magendie by insisting that he had stolen Bell’s experimental methods and findings – a significant plank of his attempts to whip up support for his priority claims. In 1824, Shaw revisited a story he had first recounted two years earlier of a trip made to Paris to promote Bell’s discoveries. The retelling was motivated by the enthusiastic reactions to Magendie’s public experiments on the facial nerves, which he had performed opposite Bell’s Great Windmill Street school in 1824. The visit would become a cause célèbre for anti-vivisectionists,^39^ but at the time Bell and Shaw were concerned about methodological and intellectual theft. Shaw was rankled by the crowds’ enthusiastic reaction:

the mass […] of spectators were so much delighted as to shake hands with each other, and congratulate themselves on having witnessed these extraordinary and *novel* facts […] but a very few […] were as much astonished that it did not seem to be known that the experiments, which were exciting so much wonder, had been repeatedly done in the rooms on the other side of the street; and that, years ago, accounts of them had been published […].^40^

He then redescribed the experiment he had conducted in front of Magendie at Alfort in 1821, implying a seamless and successful display, identical in all respects to the findings presented by Magendie in London. However, the original account revealed the extent to which Bell’s practice was fundamentally different to Wilson Philip’s and the new breed of experimental physiologists.

Shaw had visited Paris in 1821 to publicise Bell’s respiratory nervous system, particularly the role of the facial nerves in transforming the heart and lungs into a combined ‘organ of the passions’.^41^ After meeting in Paris, Magendie and Shaw travelled together to the Alfort veterinary school, where Shaw performed a public experiment on the facial nerves of a horse. The performance could hardly have been described as a success. Dividing an unspecified branch of the *fifth* on one side of the face and the *seventh* (*portio dura*) on the other, he blundered around, hacking at the facial nerves of the poor beast, failing to produce the desired experimental result, and having to get the assistance of the audience to correct his mistakes.^42^ Horses had neither been experimental nor anatomical subjects in Bell’s research programme; consequently, Shaw’s experiment foundered upon his ignorance of equine anatomy. We can only speculate what his audience made of this. We can be fairly certain, however, that as he probed, sliced, and botched, he provided a running commentary to the gathered host that bore witness to Bell’s divinely ordered respiratory nerves. We can be absolutely certain, however, that Bell’s experimental method made little or no impression upon the way that Magendie conducted his physiology, nor, indeed, upon the practices of any other experimental physiologist of note.

The Alfort episode illustrates the extent to which Bell’s programme was committed to ‘old anatomy’.^43^ Human dissection was the *sine qua non*, followed by the comparison of the human nervous system with animals placed upon the higher rungs of the ladder of creation. There was, Bell maintained, no need to carry out experiments on anything but a limited basis. ‘It is necessary only to trace the tubes, or to observe the symmetrical order of the nervous cords, in order to discover their uses’.^44^ Here, issues of practice and identity intertwined, reinforcing each other. Bell maintained the identity of the anatomist, who dissects corpses, carefully charting the course of the nerve fibres through the body, documenting their beginnings, connections, and terminations, and making preparations better to illustrate their course.

Bell placed anatomists in direct opposition to experimental physiologists, insisting that experiments had ‘never been the means of discovery’, and while a review from overseas had lauded Bell’s discoveries as a triumph for experimentation, it was nothing of the sort. His discoveries were ‘deductions from anatomy’.^45^ Those physiologists who resorted willy-nilly to vivisection misconceived ‘the just application of experiments’ to unravelling the mysteries of the nervous system. Here, he characterised brutal men as boasting ‘that they proceeded to their experiments without preparation’.^46^ ‘Preparation’ meant detailed attention to the minutiae of human and animal anatomy, combined with clinical observation, and it was for this reason that he loudly celebrated the British anatomical tradition of the Monros and the Hunters in contrast to French experimental physiology, which anyway relied on the work of anatomists like Bell for its experimental design while claiming it did not.^47^ But because Bell was an ‘old anatomist’, his discipline nevertheless demanded he perform vivisections. It was not enough to trace the passage of the nerves from brain to spinal cord to organ: experimentation served as the ultimate confirmation of careful anatomical study, ‘having taken all the assistance that the knowledge of the human structure and comparative anatomy afford, we are prepared to decide the matter by experiment’.^48^ More strongly, his insistence that when ‘the facts of anatomy’ are ‘strictly attended to, every experiment is decisive; and the truth comes out so clear and simple’ is the epitome of ‘old anatomy’.^49^ All of this was evident in the failed Alfort demonstration, which had singularly failed to ‘strictly’ attend to anatomy.

This was a far cry from the role vivisection played in experimental physiology. The ‘new physiologist’ experimented upon the nervous system in a systematic and symmetrical manner. Systematic meant removing one part of the brain, or dividing one nerve at a time, destroying the vital powers bit by bit. Symmetry required the physiologist not to divide, as a single step, the *fifth* on one side of the face and the *portio dura* on the other, to observe the effects of the combined operation – the procedure favoured by Bell. Usually, in the case of the facial nerves, the pair would be divided – left and right. Furthermore, the subject of the experiment would not survive unless further observations and experiments were to be conducted in the following days, even if the animal could viably live, as was the case when the facial nerves were the target. The animal in question would then be killed, followed by a post-mortem to ensure that the correct nerves had been divided and that the experiment had not been vitiated by collateral damage to nearby nerves.

The rigorous methods of emerging experimental physiology were visible in the practice of Britain’s two most prominent experimentalists – Wilson Philip and Marshall Hall. Mayo and Magendie, Bell’s two main competitors for priority, also followed these protocols. Magendie’s discovery of the motor and sensory roots of the spinal nerves was performed upon fully conscious puppies – an experiment that would have been morally inconceivable to Bell. That Magendie had at his disposal a litter meant he could perform every thinkable permutation of nerve division: dividing only the ventral or the dorsal root; then slicing both; and repeating the process across the litter until his interpretation was congruent with his observations.^50^ In exploring the facial nerves, Mayo’s experiments were thorough, dividing each of the nerves in question above the junction points where the nerves branched. He always divided the nerve in the same place on both the left and right sides. And he was thoroughly dismissive of Bell’s methods: according to Mayo, one of Bell’s experiments was inconclusive ‘because the nerve was not divided on both sides: had this point been attended to, a different result would probably have ensued’. Having described Bell’s key experiments, Mayo sardonically asked ‘the reader to decide’ whether the experiments were ‘satisfactory’ and bore ‘out his inferences’.^51^

In conducting his experiments on the facial and visceral nerves, Bell maintained that experiment merely offered confirmation of what anatomy had already discovered. On one level, this demonstrates the power of identity to shape the actions of its disciples. Identity functions as both a methodological and ethical framework: not only *should* you conduct research in a certain way, but you *must* also do it that way. Disciplinary commitment demands specific behaviours that, by necessity, shape the actions of its adherents, creating different imperatives for the practice of physiological investigation. In simple terms, ‘old anatomists’ insisted on limited experimentation, while new physiologists emphasised the necessity of repetition. But because ‘old anatomists’ and new physiologists vivisected, both conflicted with an increasingly influential moral economy that insisted upon treating animals humanely. This allowed for a variety of defences, including Wilson Philip’s proto-ethical code and Bell’s rhetorical efforts to distance himself from the methodical and brutal thoroughness of experimental physiology. Most importantly, both used techniques derived from slaughterhouses that aimed to mitigate the suffering of experimental subjects. While Bell may have been a strict disciple of ‘old anatomy’, like many new physiologists he was prepared to use these techniques to reconcile cruelty with humanity.

## Welcome to the slaughterhouse: vivisection in the early nineteenth century

Whether one was an ‘old’ anatomist or an experimental physiologist, the charge of cruelty remained the same, not least because it was vivisection, not a specific discipline, that was the target of reformers. As Wilson Philip confessed, he shared ‘the aversion […] which every man must feel’ to animal experimentation.^52^ This may have been so, but the public’s aversion was of a piece with other eighteenth- and nineteenth-century developments, including the persistence of ostensibly cruel practices like cock- and dog-fighting in the face of a vociferous anti-cruelty movement, although movement might be too strong a word for the disparate interests that attacked the old order of animal use.^53^ Furthermore, the increasing popularity of companion animals amongst the middling sort and the impact of sentimentalism and Romanticism more generally added to the growing sense that cruelty towards animals was morally objectionable.^54^

Medical men were not immune to these developments, which, no doubt, amplified their unease about vivisection. Most privileged other forms of investigation, particularly comparative anatomy, which was presented as an alternative to vivisection by some private school anatomists.^55^ At the same time, even those who criticised the French experimentalists did not reject vivisection as a valid technique of medical science, a perspective that became apparent after Magendie’s 1824 London visit. The debates that followed the Parliamentary interventions of Richard ‘Humanity Dick’ Martin, where he raised the spectre of Magendie in his attempts to extend anti-cruelty legislation to cover vivisection, revealed that even those who were against Magendie’s methods were in favour of limited vivisection, particularly if it could demonstrate benefit to humanity.^56^

Some historians have characterised vivisection as a ‘Frankenstein science’,^57^ but the criticisms were, in reality, focused on the pain it caused animals (often characterised as ‘torture’) and the potential brutalisation of the vivisector. Wilson Philip attempted to counter both points, and his response was significant for the influence it would have upon Bell’s experimental programme. He proposed two specific measures. One of these related to the choice of animal – where two animals were equally suitable, the ‘one which would suffer least’ should be chosen. This will be addressed once I have investigated the other measure: making the animal insensible prior to experimentation – the ultimate method of pre-anaesthetic pain reduction designed to ward off criticisms of ‘wanton’ or excessive cruelty.^58^ The animal was brought as close to death as possible. Unfeeling and unconscious, teetering on the brink between life and death, vital functions were artificially maintained using ventilators even after the removal of the animal’s head or significant parts of its brain. In making the animal nearly dead, vivisectionists used techniques employed in the abattoir and knacker’s yard, an indication of a ‘dialogue’ between the worlds of the vivisector and that of the knacker-cum-slaughterman. Ironically, this realised the rhetoric of vivisection’s opponents, who sometimes damned the former by association with the latter.^59^

The two main slaughterhouse techniques early nineteenth-century vivisectors used for making animals insensible were poleaxing and pithing, although pithing criss-crossed the boundaries between the two domains. In Britain, stunning was the most commonly employed means of slaughterhouse killing. Here, the slaughterman struck the animal’s forehead with the thimble-end of a poleaxe. If well aimed and powerful enough, a single blow left the animal unconscious, deprived of its senses and ready for a painless exsanguination.^60^ However, in the early nineteenth century, Lord Somerville, then a prominent member of the Board of Agriculture, distressed by the inefficiency of poleaxing, travelled with a companion to Lisbon, where together they learnt the art of ‘pithing’ – using a stab to divide the spine in the neck.^61^ This also appears to have been independently introduced to a Wisbech butcher by a Captain Clarkson, ‘who had seen [cattle] […] so slaughtered for the use of our fleet when at Jamaica’.^62^

Pithing was put forward as a humane method of slaughter that might replace the inefficient cruelty of poleaxing, which often led to multiple blows raining down upon the unfortunate animal’s forehead before the desired result was achieved.^63^ However, Thomas Du Gard, a physician at the Shrewsbury Infirmary, argued the contrary. Clinical evidence had led him to believe that pithing merely destroyed the sensibility below the division and did nothing to diminish the distress of the animal, just its ability to express pain. He advocated for the continuation of stunning, which at least had the merit of destroying the *‘power of feeling’.* ^64^ The anatomist Everard Home disagreed. His brother-in-law John Hunter’s papers described experiments made some years prior to Somerville’s Iberian sortie. Hunter, in tandem with Cruikshank, had shown that if the knife was ‘directed by the skill of the anatomist upwards into the cavity of the skull, so as to divide the medullary substance above the origin of the nerves which supply the diaphragm’, death would be instantaneous.^65^ The problem, then, was that some were dividing the spine below these nerves. Home also insisted that the experiments of Hunter and Cruikshank had made anatomists fully aware of the effects of pithing many years prior to Somerville’s innovation in butchery.^66^ We can only conjecture about the speed and extent to which the modified form of nape stab, as it became known, passed back into slaughterhouse practice, although it was approvingly discussed in *The Experienced Butcher* (1816). Certainly, later in the century, the poleaxe continued to be used by some and the nape stab by others.^67^ However, what this knowledge revealed to vivisectionists was that, depending upon where the stab was made, sensibility could be destroyed without killing the animal.

From what we can glean from the accounts of vivisectionists themselves, they held to Wilson Philip’s prescriptions, and they did so by poleaxing and pithing. Poleaxing was sometimes used upon the higher mammals. For example, on one of the rare occasions that Marshall Hall chose a large animal as a subject, he stunned it. ‘A horse was struck with a poll-axe [*sic*] over the anterior lobes of the brain. It fell instantly, as if struck with a thunder-bolt’, was his expressive, yet understated description. ‘It was convulsed and then remained motionless’, he continued. ‘It shortly began to breathe, and continued to breathe freely by the diaphragm’. At which point, he lacerated and pricked the horse to ensure that all sensibility had been destroyed.^68^

Often, but not always, whenever a vivisector referred to stunning, the deed was done with a poleaxe. In Bell’s case, the evidence points to a mixed practice of stunning and pithing. Shaw noted that Bell generally ‘contented himself with ascertaining’ the effects of nerve division ‘after the animal had been stunned’, while Caesar Hawkins, who acted as an assistant in many of the experiments, described animals ‘stunned or deadened to pain’.^69^ But whether or not this was poleaxing remains unclear. A former pupil, who was as ardent in his admiration of Bell as he was adamant about the necessity of vivisection, noted that in the vast majority of cases Bell’s experimental subjects were insensible,^70^ while the *London Medical Gazette* (1837) explicitly stated Bell ‘almost always’ had ‘recourse to’ dividing the spinal cord ‘just below the medulla oblongata’ – in other words a modified neck-stab, although there is only one explicit reference to its use by Bell himself.^71^ The likelihood is, as Shaw implied, that animals were poleaxed before experiments were conducted on nerves below the neck.^72^ But whatever method was used, the slaughterhouse and the anatomy theatre had come together in those of Bell’s experiments that necessitated the death of the animal. More importantly, however, Bell did not reduce his subjects to insensibility if the experiments were conducted upon the facial nerves. Here, it was crucial that the animal remained conscious so the impact upon the subject’s expression could be visually assessed.

The choice of experimental subject was an important part of the equation. This was determined by several interlocking factors, balancing the availability of subjects with the object of the investigation itself. London was teeming with animals – most working, others feral, a few for exotic display, and many companions – so unless the experimenter was determined to vivisect an exotic species, he was spoilt for choice. Rabbits were in bountiful supply on the outskirts of the city and its open heaths. Reptiles, like newts or snakes, could be found here too. Feral dogs and cats abounded. As a young man, Astley Cooper snatched ‘strays’ from the street for experimental subjects, but later paid his servant to organise a network of collectors for him.^73^ Even John Shaw once abducted a pointer.^74^

Foul-smelling knacker’s yards, found in the close and foetid streets of places like Sharp’s Alley (Farringdon), studded the city and were clogged with the entrails and hides of worn-out horses and donkeys – the beasts of burden that pulled the carriages and carts, or carried the heavy loads of the costermongers as they plied their street-side trades. Condemned cattle, warranted unfit for human consumption, were also found there. All were awaiting their fate: to be reduced to hide, glue, dyes, animal feed, or even sausages. The yards offered ample opportunities for the vivisector.^75^ But these charnel houses were not the only places to locate a subject. At the ‘Smithfield races’, held every Friday, costermongers and their ilk traded serviceable if ageing asses for a few shillings – a far cheaper prospect for the medical experimentalist than the cadavers purchased from the resurrection men.^76^

In making a choice of subject, the vivisectionist was faced with weighing the suffering caused by the experiment with the knowledge that might be gleaned. On the one hand, Wilson Philip insisted that given two equally useful animals, the one likely to suffer the least ought to have determined the choice. But on the other, if experiments were conducted to illustrate the workings of the human body, the animal needed to possess physiological characteristics that could be extended to humans. Here, comparative anatomy was essential, allowing the identification of homological nerves shared by humans and their animal subjects.^77^ At the same time, cultural values were always part of the equation. The moral worth of different species was organised in a hierarchy of sympathy that gave more credence to dog over rabbit pain, while the ‘sociozoologic scale’ granted greater ethical concern to the higher mammals over, say, the lowly frog.^78^ No doubt experimentalists like Wilson Philip believed they could offset moral outrage by choosing rabbits and frogs. But this was not an option for Bell, who was an ardent believer in natural theology combined with the Great Chain of Being.^79^ In his mind, the entire animal kingdom exhibited intentional and hierarchical design where the full range of emotions were granted to humans as a gift from God, who had also seen fit to invest the higher mammals with a limited but decreasing range of emotional expression as one descended the scale of nature. Because the expression of emotions was the *raison d’être* of his experiments, he was forced to experiment on those species that were highest on the scale of nature, meaning he was more likely to vivisect animals granted greater compassionate weight by culture than those chosen by Wilson Philip. Put simply, impassive rabbits and cold-hearted frogs would not do. Thus, there were compelling reasons for vivisecting the asses and dogs that he so loved – for only they could reveal the covert working of the respiratory nerves that were fundamental for understanding the expression of emotions.^80^

## The making of a sentimental vivisector: Charles Bell and the navigation of feeling

Any reader of Alexander Shaw’s *Narratives of the Discoveries of Sir Charles Bell* (1839) written to quell the dissent that had arisen over Bell’s claims to priority in the spinal nerves would be baffled by the space it gave to the facial nerves. The greater of the two discoveries was undoubtedly the former, while the latter has been regarded as, at most, a footnote. And yet, a single chapter was reserved for Magendie’s misdemeanours, while the rest of the book concerned itself with Mayo, who had had the temerity to ridicule Bell’s respiratory nervous system.^81^ In reality, Alexander could muster little evidence connecting Bell with experiments on the spinal nerves, because few had been reported: two for *Idea* and one for the respiratory nerves. The first was brief and understated (the species unidentified), while the latter was conducted on a stunned ass as an afterthought to experiments on the respiratory nervous system.^82^ Consequently, to claim priority over the spinal nerves, Bell had to rely upon his facial nerve experiments, particularly upon his descriptions of the *fifth*, which resembled the anatomy of the spinal nerves. To have recognised the separate motor and sensory roots of the mandibular branch of the *fifth* was to have anticipated the discovery of the separate functions of the spinal roots.^83^

But the special pleading that was at the heart of Shaw’s *Narratives* masked the real, emotions-focused, catalyst for Bell’s research, epitomised by John Shaw’s 1822 description of a series of vivisections he conducted at Bell’s behest.^84^ Shaw introduced the subject with a short discussion of clinical conditions, some of which would later be labelled ‘Bell’s palsy’, often involving distortions of laughing or sneezing faces. He then sketched out the gross and comparative anatomy of the *portio dura* (the facial portion of the *seventh* nerve) and the *fifth.* ^85^ But while the combination of anatomy and comparative anatomy led to significant deductions about the functions of these nerves, vivisections were required to confirm the results. The left facial nerve was divided ‘of the most expressive monkey’ that could be found in the Exeter Change menagerie on the Strand. The immediate effect was to destroy ‘the power of expression’ on that side. The monkey was kept alive, and for the next few weeks it was observed and occasionally goaded:

When he was irritated, he snarled and showed his teeth on the right side only […] When he is attacked with a stick, the orbicularis muscle appears to become so convulsed as to render the eye useless.^86^

The experiment was repeated on ‘several animals’ to similar effect, including dogs. In one experiment where the *portio dura* on the right side of a dog’s face was divided, the subject was ‘quite well, having suffered little from the operation; [but] when he fawns, the right side of his face is completely motionless’. On threatening the animal, Shaw observed a ‘tremulous motion expressive of fear in all the muscles of the left side of the face’, while ‘the other [side] is perfectly still; he cannot even close the eyelid, and instead of winking when he expects to be struck, the eyeball itself is turned up’. The same facial characteristics were expressed when the dog was ‘fighting with another dog’.^87^

Dogs and monkeys were not alone in having their induced paralysis antagonistically tested. Shaw divided the ‘infra-orbital’ nerve of a cat.

When she was irritated, she afforded an excellent example of paralysis of the action of the muscles regulated by this respiratory [i.e. facial] nerve. She spate [*sic*] […] with that side only, on which the nerve was entire.^88^

The *portio dura* of a game-cock was cut: ‘on opposing to another cock, there was a marked difference in the erection of the feathers of the two sides’.^89^ And so the experiments went on. An ass had its *portio dura* divided on one side, and its *fifth* on the other.^90^ Another ass was bled until unconscious, whereupon the nerves were irritated and pinched before the animal expired.^91^ An ox was exsanguinated at an abattoir where its nerves were divided, while Shaw also re-enacted an experiment on a horse’s facial nerves that he had, as we have seen, unsuccessfully attempted at the Alfort veterinary school.^92^ These experiments were all part of Bell’s exploration of his respiratory nervous system, but the facial nerve experiments were specifically designed to stimulate the subjects’ emotions – ‘expressive’ monkeys, spitting cats, angry or fawning dogs, fighting cocks.^93^

The target of these experiments may have been the emotions, but their conduct placed large emotional overheads on Bell and his subordinates. While the new ‘humane’ techniques allowed deep probing of the visceral nerves connecting heart, lungs, and face, there was little that could be done to alleviate the pain of pricking or cutting the *fifth*; and yet, without slicing the facial nerves, he could not identify the functional substrate of emotional expression. Bell’s gratitude, for example, about the lack of pain experienced by his subjects on cutting the *seventh* was freighted with pathos: the ‘experiment of cutting the […] nerve […] gave so little pain, that it was several times repeated on the ass and dog, and uniformly with the same effect’; a repetition that was far less likely to be conducted on the sensory or the visceral nerves, the latter requiring slaughterhouse techniques.^94^ But even with the *seventh*, the animal had to be restrained, the skin cut, and the nerve divided – hardly a trauma-free experience for either animal or operator.

In conducting these ostensibly cruel experiments, Bell constantly had to steer a path between the conflicting demands of his identity as an anatomist (the experiments could not be avoided for disciplinary reasons) and his humane beliefs (harming animals was morally unjustifiable). We have already explored how ‘old anatomy’ had emotional implications, but we should also note that, rather like Boddice’s experimental physiologists, these were nested within a larger moral economy. Michael Brown suggests that this was, echoing Reddy’s terminology, the Romantic emotional regime. Furthermore, his exploration of the connections between surgical identity and practice in the pre-anaesthetic era argues that Bell, along with many of his surgical contemporaries, was a subject of this regime. The Romantic surgeon, according to Brown, far from being the brutal and dispassionate ‘sawbones’ of myth, was a man of compassion who practised his craft with a high degree of concern for his patient’s pain and suffering.^95^ Brown has suggested that Bell, himself, was moulded by his brother John into a surgeon who was ‘a man of feeling’ treating his patients with kindness and compassion.^96^ Logically, this should extend to his vivisections. His compelling argument is given further weight by Bell’s integration into Romantic culture, including his profound interest in the emotions and his innate sympathy towards his experimental subjects.

Bell’s connections to Romanticism were personal, professional, and aesthetic. His paintings and sketches depicted Romantic scenes – beggars and hawkers, castle ruins and deserted landscapes.^97^ His wife noted he had a taste for the ‘benevolent’ and the ‘picturesque’: Bell, she said, ‘could not pass an old beggar or a negro; and an Irishman in rags was rather attractive’.^98^ His desk was loaded with works of a suitably, if somewhat staid, Romantic taste: ‘Standard poets, White’s Selbourne, Walton, Scott, &c.’^99^ Earlier he bought collections of poems by William Collins, Thomas Gray, and Thomas Campbell, ‘all to cheer […] [him] up’.^100^ He consulted with Wordsworth, communicated with Walter Scott, and moved in the more refined and polite circles of Romantic poetry; both Joanna Baillie and Anne Hunter were acquaintances, the eminent ‘bluestockings’ also having familial connections with Bell’s Scottish expatriate medical network through the physician-anatomist Matthew Baillie and the surgeon-anatomist John Hunter. His integration into this network may, however, have had less to do with medicine and more to do with his old friend the solicitor John Richardson.^101^ But he was not *too* refined. He could, in the right setting, appreciate the most dangerous poet of them all: ‘He had not a copy of Byron in the house, but “Childe Harold” was our guide in Italy’, said Marion.^102^ Marion’s portrait of her late husband is confirmed by his published letters, where, from his earliest time in London to the days immediately prior to his death, sentiment, sensibility, and a Romantic aesthetic remained ever-present. His unpublished correspondence reveals that these were entirely representative of his worldview.^103^

Bell’s professional and cultural connections would, then, seem to confirm Brown’s thesis. Indeed, Bell found it as troubling to perform vivisections on animals as he did capital operations on patients,^104^ and we can see in his articles and published letters the efforts he made to steer a course between disciplinary demands and his warranted moral qualms.^105^ But his connections to Romanticism indicate just how deep-seated were his feelings about cruelty to animals. Hierarchies of sympathy founded upon sentimental moral philosophy had already created the potential for white Europeans to extend their moral concern outwards to all of humanity. Romanticism went further. David Perkins has described how this played out with many of Bell’s cultural connections. The poetry of Anna Barbauld, Joanna Baillie, and Wordsworth frequently dwelt upon compassion to animals. Beyond this circle, Bell would have been well-versed in Robert Burns, particularly ‘To a Mouse’, which portrayed a ploughman extending his compassion to an animal usually regarded as vermin. Coleridge’s ‘To a Young Ass’, too, would have affectively chimed with Bell’s wider concerns about animal creation and his love of the much put-upon ‘cuddies’.^106^

I believe, however, that there were, contrary to Brown’s argument, two moral economies at play in Bell’s medical and scientific world. Sympathy, Brown’s major passbook for his Romantic regime, was central to eighteenth-century sentimentalism, which predated and prefigured Romanticism. This was not the hackneyed sentimentalism of literature, nor the canting sentimentality of the weeping dandy; rather, the sentimentalism of the empirical philosophers, particularly those of the Scottish Enlightenment, which forged strong connections between the nervous system and sympathy.^107^ Bell’s surgical compassion fitted nicely within a broadly sentimental moral economy, and his experimental programme reflected sentimentalism’s concerns. His moral concerns about the suffering to animals caused by his experiments were, however, fully Romantic – and it was this, above all else, that shaped and constrained his experimental programme.

Bell was born and brought up in the sentimental moral economy, which in its Scottish guise placed enormous weight on the relationship between sympathy and the nervous system. Edinburgh was a hotbed of thinking about nerves and the role they played in medicine and society.^108^ Christopher Lawrence highlighted Edinburgh medicine’s unwavering focus on the nervous system, which ‘had come to dominate Edinburgh physiology’, while also highlighting the connections between medicine and philosophy.^109^ Medically, three stood out, each a professor at the University: Robert Whytt (1714–66); William Cullen (1710–90); and Alexander Monro *Secundus* (1733–1817), who taught Bell. All shared an interest in nervous physiology and pathology. All were committed to experimentation. Whytt, for example, had replaced Boerhaavian mechanism with the ‘sentient principle’, a form of vitalism that imagined the soul united with the sensory nerves that would echo some of Bell’s findings. Like many of his Edinburgh peers, he was interested in how sympathy between different organs was established by the nervous system – another foundation of Bell’s nervous discoveries.^110^ The focus of the new nervous physiology was directly connected to Scottish Enlightenment philosophy. Where Whytt explored the foundations of nervous sympathy, David Hume and Adam Smith described its social expression.^111^ The philosophy of the passions, affections, and sentiments was central to Scottish Enlightenment moral philosophy, whether it was propounded by sentimentalists, rationalists, or the Common Sense philosophers, who took a position halfway between the two. Bell’s ideas and investigations, therefore, neatly fused with the priorities of Scottish Enlightenment philosophy, focusing upon critical issues around mind, memory, and emotions, often through the lens of a thorough-going Associationist. It was this combination of science and sentimental philosophy that drove his interest in the tight connections between the expression of emotions and the nervous system.^112^

Bell’s experimental investigations were, however, directly opposed to the Romantic moral economy that shaped his attitudes to animals. However much he detested causing pain, his discipline demanded he inflict it. Indeed, there was an irreconcilable gulf between his disciplinary identity and his cultural allegiances, which produced tangible effects. We might speculate, for example, that he remained first and foremost an anatomist because of his ethical qualms – his insistence upon the brutality of new physiology an emotive attempt at allaying his conscience. As Bell’s first obituarist stated: he ‘was not of the Frenchified butcherly school of anatomical experimenters’.^113^ But the strongest evidence for the effects of Bell’s rocky emotional navigation lies in his abandonment of experimentation after the death of John Shaw in 1827. Shaw was the ultimate emotional bulwark, able to undertake experiments for which Bell had no stomach. As Caesar Hawkins, sometime assistant at the Great Windmill Street school, said: ‘Sir Charles […] could not bear to witness suffering and torture, even for the sake of science, and it was not till […] Shaw, with myself or other assistants, had prepared everything for him that our chief ventured to appear and observe, and frequently not till after the animals had been stunned and deadened to pain’.^114^ Shaw’s untimely demise put a resounding full stop to Bell’s experimental programme.

The ethics of experimental physiology in the pre-Darwinian era emerged out of a similar negotiation between disciplinary identity and the sentimental moral economy. In pursuing a rigorous style of experimental physiology, Wilson Philip’s ethics were a response to increasingly vocal criticisms of humane culture. The critiques were not new – they were part and parcel of Hogarthian culture, with the longstanding belief that certain behaviours were morally corrupting. Like the profession of butchery, repeated vivisections – according to critics – hardened the operator to cruelty, vaccinating him against the sympathy that was due to the suffering subject. Wilson Philip responded by asserting his compassion, while choosing experimental subjects with less cultural cachet.^115^ He hoped to strengthen his ethical defences by employing the slaughterhouse techniques that minimised pain. But none of this could remove the taint of ‘wanton’ cruelty, and unlike Bell, Wilson Philip’s writing was gore-soaked, even as he protested his humanity. The ethical system he proposed was driven, like Bell’s vacillations, by an acknowledgement that what he did opposed cultural norms. But this, he believed, should be offset by weighing the everyday cruelty suffered by animals (for work, sport, or food) against vivisection’s potential benefits for humanity. The only solace Bell could find was in the limited experimentation of ‘old anatomy’, but even then, his conscience would not be fully salved.^116^

## Conclusion: manliness and vivisection from sentimentalism to Darwinism

This case study of Bell’s experimental practices reveals the tensions between the anatomy theatre and the slaughterhouse. Bell’s simultaneous love for animals and dependence upon their experimental destruction created an ethical dilemma that he never fully resolved. Comparing Bell and Wilson Philip demonstrates distinct approaches to the same moral predicament. Whilst Philip developed systematic protocols to minimise animal suffering through slaughterhouse techniques and a reliance on animals set lower on the sociozoological scale, Bell’s experiments on the facial nerves required the observation of conscious subjects high on that scale to observe the relationship between the nerves and emotional expression. This rendered many of Wilson Philip’s innovations inapplicable to his most important work, forcing him to navigate between disciplinary obligations and personal ethics, shaping his navigation between scientific duty and the nested economies of sentimentalism and Romanticism.

Part of this process relied upon specific cultural understandings of how character operated as a defence against culturally abhorrent practices like vivisection. As we have seen, the arguments made in favour of the practice balanced the benefits to humanity with compassion. There was also the need to exercise constant vigilance against the moral corruption of brutal and brutalising practices. Just as in pre-anaesthetic surgery, the protection lay in the character of the operator, who had to shape an appropriate emotional response to the suffering he inflicted. This was achieved through resources provided by specific moral economies, while mobilising a particular form of manliness that guaranteed the probity of the vivisectionist as an upright moral being. In this, Bell was far more successful than Wilson Philip, who, despite the importance of his findings, has been largely forgotten. Bell could at least mobilise the persona of ‘a gentleman and a Christian’.^117^ This was, however, a fundamentally different form of manliness to that which acted as a bulwark for experimental physiologists during the 1870s, the years of its greatest controversy. The scale of nature had been replaced by Darwinian common descent, and anaesthetics had made slaughterhouse techniques redundant. The compassionate economy that had underpinned Bell’s navigation of feeling and determined Wilson Philip’s rudimentary ethics had been displaced by a Darwinian economy where ethics were underwritten by a different incarnation of character that emphasised stoicism and *sang froid.* ^118^

## Footnotes

1 See, for example, Paul F. Cranefield , The Way in and the Way Out. François Magendie, Charles Bell and the Roots of the Spinal Nerves (Mount Kiskco, NY: Futura Publishing, 1974); Pauline Mazumdar , ‘Anatomy, Physiology, and Surgery: Physiology Teaching in Early Nineteenth-Century London’, Canadian Bulletin of Medical History, 4, 1 (1987), 119–4311612338 10.3138/cbmh.4.2.119; Gillian Rice , ‘The Bell-Magendie-Walker Controversy’, Medical History, 31, 2 (1987), 190–200 3550329 10.1017/s0025727300046561PMC1139711; James Bradley , ‘Matters of Priority: Herbert Mayo, Charles Bell and Discoveries in the Nervous System’, Medical History,58, 4 (2014), 564–8425284895 10.1017/mdh.2014.53PMC4176267; Carin Berkowitz , Charles Bell and the Anatomy of Reform (Chicago: University of Chicago Press, 2015), 130–64.

2

James Bradley , ‘Redefining a Discovery: Charles Bell, the Respiratory Nervous System and the Birth of the Emotions’, Studies in the History and Philosophy of Science, 106 (2024), 12–20 10.1016/j.shpsa.2024.04.00338850832.

3

Charles Bell , An Exposition of the Natural System of the Nerves of the Human Body, with a Republication of the Papers Delivered to the Royal Society (London: A. & R. Spottiswoode, 1824), 2–3 ; Berkowitz, *op. cit.* (note 1), 142–51; his humanity and reluctance to vivisect are noted by his biographers, including the two earliest: Anon. [Robert Ferguson], ‘Article VII’, *Quarterly Review*, 72, 143 (1843), 192–231, 204; and, Amédée Pichot , The Life and Labours of Sir Charles Bell (London: Richard Bentley, 1860), 68 .

4 See John Shaw , ‘On the Difference of the Functions of Certain Nerves of the Face, Illustrated by Their Anatomy in the Inferior Animals, and by a Comparison of Their Uses in Man and Brutes’, The Quarterly Journal of Science, Literature and Art, 12, 24 (1822), 231–43.

5 Anon., *Letters of Sir Charles Bell From His Correspondence With His Brother George Joseph Bell* (London: John Murray, 1870), 265 (drawing donkeys), 399 (day before death); also see graphite drawing, ‘Two Donkeys Browsing; Two Donkeys in a Field With Trees’, British Museum, 1887, 0312.7, https://www.britishmuseum.org/collection/object/P_1887-0312-7.

6

Charles Bell , ‘On the Nerves; Giving an Account of Some Experiments on Their Structure and Functions, Which Lead to a New Arrangement of the System’, Philosophical Transactions of the Royal Society of London, 111 (1821), 398–424, 412–3.

7
*Ibid.*, 418.

8

Anita Guerrini , Experimenting With Humans and Animals: From Galen to Animal Rights (Baltimore: Johns Hopkins University Press, 2003), 75 .

9

Charles Bell , An Essay on the Forces Which Circulate the Blood: Being an Examination of the Difference of the Motions of Fluids in Living and Dead Vessels (London: Longman, 1819), 25–31 ; Charles Bell , ‘Observations on the Proper Method of Studying the Nervous System’, in Report of the Fourth Meeting of the British Association for the Advancement of Science (London: John Murray, 1835), 667–70.

10

Andrew Cunningham , ‘The Pen and the Sword: Recovering the Disciplinary Identity of Physiology and Anatomy Before 1800: I: Old Physiology – the Pen’, Studies in History and Philosophy of Science Part C, 33, 4 (2002), 631–65; Cunningham, ‘The Pen and the Sword: Recovering the Disciplinary Identity of Physiology and Anatomy before 1800: II: Old Anatomy – the Sword’, *Studies in History and Philosophy of Science Part C*, 34, 1 (2003), 51–76.

11

R.D. French , Antivivisection and Medical Science in the Victorian Era (Princeton, NJ: Princeton University Press, 1975), 20–1; Diana Manuel , ‘Marshall Hall (1790–1857): Vivisections and the Development of Experimental Physiology’, in Nicolaas A. Rupke (ed.), Vivisection in Historical Perspective (Beckenham: Croom Helm, 1987), 78–104, on 78; A.W.H. Bates , Anti-Vivisection and the Profession of Medicine in Britain – A Social History (London: Palgrave Macmillan, 2017), 17 30048075.

12

William Reddy , The Navigation of Feeling: A Framework for the History of Emotions (Cambridge: Cambridge University Press, 2001). Rob Boddice , The Science of Sympathy: Morality, Evolution and Victorian Civilization (Urbana: University of Illinois Press, 2016), 13–20 , borrows Lorraine Daston’s version of ‘moral economies’ (distinct from E.P. Thompson’s formulation), which combined ‘thought collectives’ and ‘emotion collectives’, providing a more direct understanding of how identity shapes practice.

13 Reddy, *op. cit.* (note 12), 96–110.

14 Boddice, *op. cit.* (note 12); Paul White , ‘The Experimental Animal in Victorian Britain’, in L. Daston and G. Mitman (eds.), Thinking With Animals: New Perspectives on Anthropomorphism (Chichester, West Sussex: Columbia University Press), 59–82, on 74.

15

Edwin Clarke and L.S. Jacyna , Nineteenth-Century Origins of Neuroscientific Concepts (London: University of California Press, 1987), 9–28 .

16

Ruth Richardson , Death, Dissection and the Destitute, rev. edn (Chicago: Chicago University Press, 2000); Helen MacDonald , Human Remains: Episodes in Human Dissection (Melbourne: Melbourne University Press, 2005).

17

Rob Boddice , A History of Attitudes and Behaviours Toward Animals in Eighteenth- and Nineteenth-Century Britain: Anthropocentrism and the Emergence of Animals (Lewiston, NY: Edwin Mellen Press, 2009); David Perkins , Romanticism and Animal Rights, 1790–1830 (Cambridge: Cambridge University Press, 2003); John Simons , Animal Rights and the Politics of Literary Representation (Basingstoke: Palgrave, 2002).

18

A.P.W. Philip , An Experimental Inquiry into the Laws of the Vital Functions, 3rd edn (London: Thomas and George Underwood, 1826), xlii–liii , reveals he wrote to the *New Monthly Magazine* as *Philanthropos*, countering a correspondent’s accusations of cruelty by vivisectors. The correspondence was covered in the *New Monthly Magazine*, 3–6, 18–32 (1815–16), with Philip’s letter 6, 32 (1816), 106–8.

19 Cunningham, *op. cit.* (note 10).

20

John Lesch , Science and Medicine in France: The Emergence of Experimental Physiology (Cambridge, MA: Harvard University Press, 1984); Paul Elliot, ‘Vivisection and the Emergence of Experimental Physiology in France’, in Rupke, *op. cit.* (note 11), 48–77.

21 Lesch, *ibid.*, 80–98.

22

Gerald L. Geison , Michael Foster and the Cambridge School of Physiology: The Scientific Enterprise in Late Victorian Society (Princeton: Princeton University Press, 1978), 20 (‘practitioners so few’); Bates, *op. cit.* (note 11), 17 (‘proverbially rare’).

23 Geison, *ibid.*, 43.

24

A.P.W. Philip , An Analysis of the Malvern Waters (Worcester: J. Tymbs and W. Smart, 1805).

25

A.P.W. Philip , Treatise on Febrile Diseases, Including Intermitting, Remitting, and Continued Fevers, Eruptive Fevers; Inflammations, Hemorrhagies, and the Profluvia, 3 vols (Winchester: Robbins, 1799).

26

A.P.W. Philip , ‘Some Positions Respecting the Influence of the Voltaic Battery in Obviating the Effects of the Division of the Eighth Pair of Nerves’, Philosophical Transactions of the Royal Society of London, 112 (1822), 22–3.

27

Charles Bell , Idea of a New Anatomy of the Brain (London: Strahan and Preston, 1811).

28

A.P.W. Philip , ‘Experiments Made With a View to Ascertain the Principle on Which the Action of the Heart Depends, and the Relation Which Subsists Between That Organ and the Nervous System’, Philosophical Transactions of the Royal Society of London, 105 (1815), 65–90 ; ‘Some Additional Experiments and Observations on the Relation Which Subsists Between the Nervous and Sanguiferous Systems’, *Philosophical Transactions of the Royal Society of London*, 105 (1815), 424–46; ‘On the Effects of Galvanism in Restoring the Due Action of the Lungs’, *Philosophical Transactions of the Royal Society of London*, 107 (1817): 22–31; *An Experimental Inquiry into the Laws of the Vital Functions* (London: Thomas and George Underwood, 1817).

29
*Ibid.*, ‘Experiments Made With a View’, 67.

30 Philip, *Experimental Inquiry* (note 28), 69.

31
*Ibid.*, 70–1.

32

A.P.W. Philip , ‘Dr Wilson’s *Reply to Some Observations Relating to His Inquiry into the Laws of the Vital Functions in the Last Number of the Quarterly Journal, in a Letter Addressed to W.T. Brande, Esq.*
’, The Quarterly Journal of Literature, Science and the Arts,7, 14 (1819), 349–54, 351.

33 Philip, *Experimental Inquiry* (note 28), 113.

34
*Ibid.*, 88–90.

35

James Elwick , Styles of Reasoning in the British Life Sciences: Shared Assumptions, 1820–1858 (London: Chatto & Pickering, 2007).

36
*Ibid.*, 44–5.

37 Lesch, *op. cit.* (note 20), 86–9, on Legallois as a founder of experimental physiology.

38 Bichat’s division of the nervous system is similar to Bell’s, including elements of the respiratory nervous system that seemed to have been lifted from Bichat’s work, including the location of the passions in ‘organic life’, *ibid.*, 62. Nevertheless, Bell barely acknowledged Bichat.

39 French, *op. cit.* (note 11), 26; Bates, *op. cit.* (note 11), 15–17; Guerrini, *op. cit.* (note 8), 81–4.

40

John Shaw , ‘Remarks on M. Magendie’s Late Experiments upon the Nerves’, London Medical and Physical Journal, 52, 2 (1824), 95–104 PMC563538230494696, on 96–7.

41

Charles Bell , ‘Of the Nerves Which Associate the Muscles of the Chest, in the Action of Breathing, Speaking, and Expression. Being a Continuation of the Paper on the Structure and Functions of the Nerves’, Philosophical Transactions of the Royal Society of London, 112 (1822), 284–312, on 310; Bradley, *op. cit.* (note 2).

42 Shaw, *op. cit.* (note 4), 238.

43 Both Bell and Shaw believed that understanding the respiratory nerves realised practical clinical applications including the description of the clinical signs of Bell’s Palsy; John Shaw , ‘On the Effects Produced on the Human Countenance by Paralysis of the Different Systems of Facial Nerves’, Quarterly Journal of Science Literature & Arts, 13, 25 (1822), 120–35; Charles Bell , Appendix to the Papers on the Nerves Republished From the Royal Society’s Transactions (London: Longman, Rees, Orme, Brown, and Green, 1827), later integrated into *The Nervous System of the Human Body* (London: Longman, Rees, Orme, Brown, Green, & Longman, 1830); Charles Bell , ‘On the Nerves of the Face; Being a Second Paper on That Subject’, Philosophical Transactions of the Royal Society 119 (1829), 317–330 , particularly 317–8.

44

Charles Bell , ‘Second Part of the Paper on the Nerves of the Orbit’, Philosophical Transactions of the Royal Society, 113 (1823), 289–307 , on 302.

45
*Ibid.*, 302.

46

Charles Bell , ‘On the Nervous System’, Philosophical Transactions of the Royal Society, 130 (1840), 245–54, on 247.

47 Bell, *op. cit.* (note 44), 306–7.

48 Bell, *op. cit.* (note 6), 412.

49 Bell, *op. cit.* (note 3), 2–3.

50

François Magendie , ‘Expériences sur les Fonctions des Racines des Nerfs Rachidiens’, Journal de Physiologie Expérimentale et de Pathologie, 2 (1822), 276–9.

51

Herbert Mayo , Anatomical and Physiological Commentaries, No. 2 (London: Thomas and George Underwood, 1823), 119–20.

52 Philip, *Experimental Inquiry*, *op. cit.* (note 28), xiii–ix.

53 See French, *op. cit.* (note 11), 18–20; Hilda Kean , Animal Rights: Political and Social Change in Britain Since 1800 (London: Reaktion, 1998), 13–38, 96–8; Guerrini, *op. cit.* (note 8), 70–8. The motivations of anti-cruelty campaigners were disparate and included evangelical concerns about corrupting the soul, sentimental philosophy that extended sympathy to all creation, and class-based attacks on working-class practices – see Harriet Ritvo , The Animal Estate: The English and Other Creatures in the Victorian Age (Cambridge, MA: Harvard University Press, 1987), 125–66.

54

Keith Thomas , Man and the Natural World: Changing Attitudes in England, 1500–1800 (New York: Pantheon, 1983), 110–20; Perkins, *op. cit.* (note 17), 44–65.

55

A.W.H. Bates , ‘Vivisection, Virtue Ethics, and the Law in 19th-Century Britain’, Journal of Animal Ethics, 4, 2 (2014), 30–44 ; See, in particular, Michael Ryan quote (1835) in Adrian Desmond , The Politics of Evolution: Morphology, Medicine, and Reform in Medical London (London: Chicago University Press, 1992), 183 .

56 French, *op. cit.* (note 11), 20; Guerrini, *op. cit.* (note 8), 77, for Martin’s intervention in parliament. Martin’s speech is one of the set pieces of animal rights activism that tends to drown out equivocation around vivisection. *Hansard* (11 and 24 March 1825) reveals a nuanced debate, with some defending Magendie’s character. Bates, *op. cit.* (note 11), 17–18, suggests medicine rejected vivisection outright, but the evidence he uses from the *Morning Chronicle*’s report of the SPCA (30 June 1825, 3), reveals similar levels of equivocation from medical witnesses, including Everard Home and Bell himself.

57

Anita Guerrini , ‘Animal Experiments and Antivivisection Debates in the 1820s’, in Jane R. Goodall and Christina Knellworth (eds), Frankenstein’s Science: Experimentation and Discovery in Romantic Culture, 1780–1830 (Farnham: Ashgate, 2008), 71–86 .

58 Philip, *Experimental Inquiry*, *op. cit.* (note 28), xi–xii. Ironically, during the early nineteenth century, manufactured gasses were tested on animals. In the early days of chloroform, animals were also used as testbeds. See Stephanie J. Snow , Blessed Days of Anaesthesia: How Anaesthetics Changed the World (Oxford: Oxford University Press, 2008), 11–19 and 48–9.

59 Anon., *The Voice of Humanity: Observations on a Few Instances of Cruelty to Animals* (London: Sherwood & Co., 1827), 13, bridging a section between the horrors of the slaughterhouse and those of physiology, ‘we are reminded of […] the physiological butchers’; see also Anon., ‘Experiments on Living Animals’, *London Medical Gazette*, 20, 508 (1837), 804–8, on 808.

60

Ian MacLachlan , ‘Humanitarian Reform, Slaughter Technology, and Butcher Resistance in Nineteenth-Century Britain’, in Paula Young Lee (ed.), Meat, Modernity, and the Rise of the Slaughterhouse (Lebanon, NH: University of New Hampshire Press, 2008), 107–20, 112 details the methods in use during the mid-to-late nineteenth century.

61

Joseph Plymley , General View of the Agriculture of Shropshire (London: Sherwood, Neely and Jones, 1803), 244 .

62
*Ibid.*, 244–5.

63

Mr Marshall , Review of the Reports to the Board of Agriculture From the Western Department of England (York: Longman, Hurst, Rees, and Orme), 255 .

64 Plymley, *op. cit.* (note 61), 246–50.

65
*Ibid.*, 250–6 produces a letter from Home to Lord Carrington about slaughterhouse techniques and Hunter.

66
*Ibid.*, 255.

67 Anon., *The Experienced Butcher* (London: Darton, Harvey and Darton, 1816), 142; Maclachlan, *op. cit.* (note 60), 115.

68

Marshall Hall , Lectures on the Nervous System and Its Diseases (Philadelphia, PA: E.L. Carey & A. Hart, 1836), 28–9.

69

John Shaw , ‘An Account of Some Experiments on the Nerves; by M. Majendie With Some Observations’, The London Medical and Physical Journal, 48, 4 (1822), 343–52, on 346.

70

George F. Etherington , Vivisection Investigated and Vindicated (Edinburgh: P. Rickard, 1842), 185 .

71 Anon., ‘Experiments on Living Animals’, *op. cit.* (note 59), 807; Bell, *op. cit.* (note 44), 298.

72 Shaw, *op. cit.* (note 4), 239.

73

Bransby Blake Cooper , The Life of Sir Astley Cooper, Bart: Interspersed With Sketches From His Note-Books of Distinguished Contemporary Characters (London: J.W. Parker, 1843), 142 and 334–6PMC509831129918831.

74 Cranefield, *op. cit.* (note 1), section 1811a, contains a copy of a letter from a surgeon suggesting one of his pupils ask, ‘Shaw if he remembers the experiment we performed upon on a pointer dog in Leicester Street, which he stole’.

75 Chris Otter, ‘Civilizing Slaughter: The Development of the British Public Abattoir, 1850–1910’, in Lee, *op. cit.* (note 60), 89–106, 90–2 points out that the definition of ‘slaughterhouse’ was broad, covering any dwelling where slaughter took place. For a near contemporary description of Sharp’s Alley, Richard H. Horne, ‘The Cattle-Road to Ruin’, *Household Words*, 1 (29 June 1850), 325–30.

76 Henry Mayhew, *London Labour and the London Poor; Cyclopaedia of Those That Will Work, Those That Cannot Work, and Those That Will Not Work*, Vol. 1, *The London Street Folk* (London: Griffin, Bohn, and Company, 1861), 27–9.

77

L.S. Jacyna , ‘Principles of General Physiology: The Comparative Dimension to British Neuroscience in the 1830s and 1840s’, Studies in History of Biology, 7 (1984), 74–92 11611372 counters the alleged backwardness of British physiology, arguing comparative anatomy was an effective alternative research programme.

78

Asha Hornsby , ‘Unfeeling Brutes? The 1875 Royal Commission on Vivisection and the Science of Suffering’, Victorian Review, 45, 1 (2019), 97–115 ; Arnold Arluke and Clinton Sanders , Regarding Animals (Philadelphia, PA: Temple University Press, 1996), 167–86.

79 Bell actively promoted Paley’s natural theology. See Charles Bell , The Hand: Its Mechanism and Vital Endowments, as Evincing Design (London: W. Pickering, 1834); Charles Bell and Henry Brougham (eds.), William Paley, Paley’s Natural Theology With Illustrative Notes, by Henry, Lord Brougham and Sir Charles Bell to Which Are Added Supplementary Dissertations, by Sir Charles Bell, 2 vols (London: Charles Knight, 1836); Desmond, *op. cit.* (note 55), 111–2 and 260, reveals he was sometimes ridiculed in the medical press for this commitment.

80 Bradley, *op. cit.* (note 2).

81

Alexander, Shaw , Narrative of the Discoveries of Sir Charles Bell in the Nervous System (London: Longman, Orme, Brown, Green and Longmans, 1839).

82 Bell, *op. cit.* (note 27), 21–2; Shaw, *op. cit.* (note 69), 346–7, with footnote on 346 indicating Bell had only conducted one or two experiments on conscious animals and was unwilling to repeat them.

83 Shaw, *ibid.*, 348; Mayo, *op. cit.* (note 51), 9–10.

84 Shaw, *op. cit.* (note 4), 232.

85
*Ibid.*, 232–5.

86
*Ibid.*, 235–6.

87
*Ibid.*, 237.

88
*Ibid.*, 238.

89
*Ibid.*, 240.

90
*Ibid.*, 238–9.

91
*Ibid.*, 238.

92
*Ibid.*, 238–9.

93 Bell’s use of antagonism in experimental contexts prefigures those used in the 1930s; see O.E. Dror , ‘Fear and Loathing in the Laboratory and Clinic’, in F.B. Alberti (ed.), Medicine, Emotion and Disease, 1700–1950 (Houndmills: Palgrave Macmillan, 2006), 125–43, on 132–4 for ‘animal-as-stimulus’.

94 Bell, *op. cit.* (note 6), 413.

95

Michael Brown , ‘Surgery, Identity and Embodied Emotion: John Bell, James Gregory and the Edinburgh “Medical War”’, History, 104 (2019), 19–41 30828105 10.1111/1468-229X.12720PMC6378620; Brown, ‘Wounds and Wonder: Emotion, Imagination and War in the Cultures of Romantic Surgery’, *Journal for Eighteenth-Century Studies*, 43, 2 (2020), 239–59; Brown, *Emotions and Surgery in Britain, 1793–1912* (Cambridge: Cambridge University Press, 2022), 77–82.

96 Brown, ‘Surgery, Identity and Embodied Emotion’, *ibid.*

97

Ludmilla Jordanova , ‘The Representation of the Human Body: Art and Medicine in the Work of Charles Bell’, in Brian Allen (ed.), Towards a Modern Art World (New Haven, CT: Yale University Press, 1995), 79–94, 79–80, is concerned with Bell’s medical illustrations and his *The Anatomy of Expression* (1806). Bell is celebrated for his medical illustrations and also his paintings of the survivors from the Peninsular Campaign and Waterloo. However, he took his sketch pad, pens, and other artistic materials almost everywhere he went. The paintings held in the British Museum collection are indicative of his non-medical, romantic artistic work.

98 Bell, *op. cit.* (note 6), 415.

99
*Ibid.*, 419–20.

100 Anon., *op. cit.* (note 5), 255, letter dated 19 Aug. 1816; Bell met Campbell on several occasions, having been introduced to him by John Richardson at a dinner party that included Walter Scott and Thomas Campbell, 150, letter dated 27 May 1809.

101 Joanna Baillie intervened on Bell’s behalf when he was canvassing for the Professorship of Anatomy at the Royal Academy in 1824, which he had first attempted to attain in 1807. Baillie wrote to Sir George Beaumont, who was well connected in Academy circles, asserting that not only was he eminently qualified for the position, but also had ‘re(re)quested me to do so; he is a Gentleman whom I consider myself obliged to’, Joanna Baillie to Sir George Beaumont Bart., 22 Dec. 1824, in Judith Bailey Slagle (ed.), The Collected Letters of Joanna Baillie. Electronic *Edition*, vol. 2 (Charlottesville: Fairleigh Dickinson University Press, 1999), 1167 ; on 1196, Baillie wrote directly to Bell in 1833 thanking him for a volume sent to her and her sister that including remarks on William Hunter and her brother Matthew Baillie; see also letter to Marion Bell, dated at some time between 1826 and 1831, Thomas McLean (ed.), Further Letters of Joanna Baillie (Madison: Fairleigh Dickinson University Press, 2010), 114 . For Richardson’s literary connections, see his entry in the *DNB*, W. F. Rae, ‘Richardson, John (1780–1864)’, rev. H.C.G. Matthew , Oxford Dictionary of National Biography (Oxford: Oxford University Press, 2004), http://www.oxforddnb.com/view/article/23567 ; Richardson’s poems were published in Joanna Baillie (ed.), *Collection of Poems, Chiefly Manuscript and From Living Authors* (London: Longman, Hurst, Rees, Orme, and Brown, 1823), 84 and 212. Other contributors included Scott, Southey, Wordsworth, Anne Hunter, Baillie’s own work, and a host of amateur poets.

102 Bell, *op. cit.* (note 6), 420.

103 See, for example, letter of August 1841 to Richardson shortly before his death that charts a journey he made from Hooton (Cheshire) to Rawcliffe (Lancashire) then to Belfast, Richardson of Kirklands Collection, National Library of Scotland, 3989, ff 12–13.

104

Charles Bell , A System of Operative Surgery, Founded on Anatomy, 2nd ed., Vol. 1 (London: Longman, Hurst, Rees, Orme, and Brown, 1814), vii–ix , provides the clearest example of Bell’s navigation of feeling in the operating theatre, indeed the entire work was geared towards helping ‘the young surgeon in entering into […] communing with himself’. Bell’s letters provide further insights, e.g. Anon., *op. cit.* (note 5), 281 describes ‘an anxious operation on my spirits tonight’, which he subsequently revealed went badly: ‘had a most miserable time […] from the failure of an operation, and the death of a most worthy man. I shall regret it as long as I live’. His journal, which has not survived, also recorded his impression of fellow practitioners, which seemed to echo his own navigation, Anon., *op. cit.* (note 3), 196, Astley Cooper ‘has nothing of that tormenting anxiety for his patients which becomes the burthen of life to other men’, while Abernethy ‘felt the duties of his profession a torture’.

105 Bell emphasised the psychological pain that experiments caused him, a factor that appears in both Anon., *op. cit.* (note 5), 275–6 and Anon., *op. cit.* (note 3), 204 and 211. He might admit to his brother that he is deferring experiments due to their cruelty but the experiments still happened. Bell and his various biographers emphasised the need to undertake vivisections while also portraying the psychological hurt it caused. Charles Bell , ‘On the Nerves of the Face; Being a Second Paper on That Subject’, Philosophical Transactions of the Royal Society of London, 119 (1829), 317–30, on 327–8 charted the same course of navigation for John Shaw, who conducted ‘experiments which in the gentleness of his nature he would have hesitated to make from their severity, but for their imperatively being called for’.

106 All of these poets are discussed throughout Perkins, *op. cit.* (note 17).

107

Markman Ellis , The Politics of Sensibility: Race, Gender and Commerce in the Sentimental Novel (Cambridge: Cambridge University Press, 1996), 35 .

108 Christopher Lawrence, ‘Medicine as Culture: Edinburgh and the Scottish Enlightenment’ (PhD, University of London, 1984); Christopher Lawrence , ‘Ornate Physicians and Learned Artisans: Edinburgh Medical Men, 1726–1776’, in W.F. Bynum and Roy Porter (eds), William Hunter and the Eighteenth-Century Medical World (Cambridge: Cambridge University Press, 1985), 154–76.

109

Christopher Lawrence , ‘The Nervous System and Society in the Scottish Enlightenment’, in Barry Barnes and Steven Shapin (eds), Natural Order: Historical Studies of Scientific Culture (London: Sage, 1979), 19–40 , on 24.

110 Lawrence, ‘Medicine as Culture’, *op. cit.* (note 108), 133–59.

111 Lawrence, *op. cit.* (note 110), 31–4.

112 Bradley, *op. cit.* (note 2).

113 Anon., *op. cit.* (note 3), 211.

114

Caesar Henry Hawkins , ‘Annual Address Delivered Before the Fellows of the Royal Medical and Chirurgical Society, on Saturday, March 1st, 1856’, Lancet, 1, 1698 (1856), 283 .

115 Philip, *Experimental Inquiry*, *op. cit.* (note 28), ix–xi; he strengthened his statements about cruelty further for the 3rd edition of *Experimental Inquiry*, *op. cit.* (note 18), xliii–liii.

116 Anon., *op. cit.* (note 5), 275–6.

117 Anon., *op. cit.* (note 3), 211.

118 Different models of manliness are central to the work of both Boddice and Brown. See, for example, Boddice, *op. cit.* (note 12), 44 and Brown, ‘Surgery, Identity and Embodied Emotion’, *op. cit.* (note 95), 38; White, *op. cit.* (note 14), 74. The 1870s ethics of vivisection, described by Boddice and White, present a fundamentally different moral economy to the sentimental, derived from a combination of utilitarianism and, according to Boddice, Darwinian materialism. Unlike David Ferrier, Bell never defended his use of vivisection by referring to his love of dogs. Nor did he ever make the argument that his willingness to vivisect demonstrated that he was more compassionate than anti-vivisectionists.

